# Analysis of a *Clostridium difficile* PCR ribotype 078 100 kilobase island reveals the presence of a novel transposon, Tn*6164*

**DOI:** 10.1186/1471-2180-12-130

**Published:** 2012-07-02

**Authors:** Jeroen Corver, Dennis Bakker, Michael S M Brouwer, Céline Harmanus, Marjolein P Hensgens, Adam P Roberts, Len J A Lipman, Ed J Kuijper, Hans C van Leeuwen

**Affiliations:** 1 Department of Medical Microbiology, Section Experimental Microbiology, Center of Infectious Diseases, Leiden University Medical Center, Leiden, The Netherlands; 2 Division of Microbial Diseases, UCL Eastman Dental Institute, University College London, London, UK; 3 Division of Veterinary Public Health, Faculty of Veterinary Medicine, Institute of Risk Assessment Sciences, Utrecht University, Utrecht, The Netherlands; 4 LUMC, Medical Microbiology, E4P, Postbus 9600, 2300 RC Leiden, The Netherlands

**Keywords:** *Clostridium difficile*, Transposable element, Phage, Antimicrobial resistance, Virulence

## Abstract

**Background:**

*Clostridium difficile* is the main cause of antibiotic associated diarrhea. In the past decade, the number of *C. difficile* patients has increased dramatically, coinciding with the emergence of two PCR ribotypes 027 and 078. PCR ribotype 078 is also frequently found during *C. difficile* outbreaks in pigfarms. Previously, the genome of the PCR ribotype 078 strain M120, a human isolate, was described to contain a unique insert of 100 kilobases.

**Results:**

Analysis of this insert revealed over 90 open reading frames, encoding proteins originating from transposons, phages and plasmids. The insert was shown to be a transposon (Tn*6164*), as evidenced by the presence of an excised and circularised molecule, containing the ligated 5’and 3’ends of the insert. Transfer of the element could not be shown through filter-mating experiments. Whole genome sequencing of PCR ribotype 078 strain 31618, isolated from a diarrheic piglet, showed that Tn*6164* was not present in this strain. To test the prevalence of Tn*6164*, a collection of 231 *Clostridium difficile* PCR ribotype 078 isolates from human (n = 173) and porcine (n = 58) origin was tested for the presence of this element by PCR. The transposon was present in 9 human, tetracycline resistant isolates, originating from various countries in Europe, and none of the pig strains. Nine other strains, also tetracycline resistant human isolates, contained half of the transposon, suggesting multiple insertion steps yielding the full Tn*6164*. Other PCR ribotypes (n = 66) were all negative for the presence of the transposon. Multi locus variable tandem repeat analysis revealed genetic relatedness among transposon containing isolates. Although the element contained several potential antibiotic resistance genes, it did not yield a readily distinguishable phenotype.

**Conclusions:**

Tn*6164* is a newly described transposon, occurring sporadically in *C. difficile* PCR ribotype 078 strains. Although no transfer of the element could be shown, we hypothesize that the element could serve as a reservoir of antibiotic resistance genes for other bacteria. Further research is needed to investigate the transfer capabilities of the element and to substantiate the possible role of Tn*6164* as a source of antibiotic resistance genes for other gut pathogens.

## Background

Over the past decade, *Clostridium difficile* has emerged as an important gut pathogen, causing hospital- and community-acquired diarrhea. The number of patients and the severity of disease have increased dramatically, due to the emergence of two hypervirulent PCR ribotype, 027 [1] and 078 [2,3]. Traditionally, PCR ribotype 027 has been linked to nosocomial outbreaks. In contrast, PCR ribotype 078 has been detected frequently in farming animals, especially pigs [2,4], and is found more during community acquired infection. The increase in *C. difficile* infections (CDI) of humans has boosted interest in *C. difficile* biology, diagnostics and pathogenesis.

In the past few years, multiple genome sequences of several PCR ribotypes have been determined [5-8]. The analyses of the genomes, aided by comparative genomics of DNA-DNA microarrays [9,10] has shown that the genomes of *C. difficile* are highly variable with inserts of mobile DNA from phage, plasmid or transposon origin. These mobile DNA elements are actively moving within *C. difficile* genomes and are frequently passed on to neighboring bacteria, harboring mosaic genomes [7,11]. It is unclear what role the mobile elements play in the virulence of *C. difficile.* Some virulence linked genes, for example the holin-like *tcdE*, have a phage origin [12]. In fact, it has been suggested that the whole pathogenicity locus (PaLoc), encoding the major *C. difficile* virulence factors TcdA and TcdB, is of phage origin [13,14]. Recently, phages have been shown to upregulate toxin production in *C. difficile*, thereby increasing the virulence [15]. *C. difficile* transposons have been shown to contain antibiotic resistance genes [5,7,16,17], and therefore acquiring such an element could increase the virulence and/or colonization potential of a particular strain.

Mobile elements play an important role in the diversification of bacterial genomes. One important group of mobile genetic elements is the Tn*916* family of conjugative transposons (also known as integrative and conjugative elements [ICEs]) [18]. These conjugative transposons usually code for tetracycline resistance and are found primarily in the *Firmicutes*. Numerous transposons have been described to be present in *C. difficile* genomes [5,7,11,17,19]. Several elements closely related to Tn*916* are present in diverse *C. difficile* strains, including Tn*5397* which confers tetracycline resistance [20,21]. Other transposons have been described to confer resistance to chloramphenicol and erythromycin [5].

Recently, the first full length genome of a PCR ribotype 078 strain was published [5]. This M120 strain has been isolated from an Irish diarrheic patient. It was shown that PCR ribotype 078 is highly divergent from PCR ribotype 027, 001, 017 and 012. In addition, this PCR ribotype 078 strain was described to contain a unique 100 kb insert that showed 80% similarity to sequences of *Thermoanaerobacter* species and *Streptococcus pneumoniae*[5]. In this paper we show that the 100 kb insert is a mobile element that is only sporadically present in PCR ribotype 078 strains. Furthermore, we show that the 100 kb consists of at least two independent mobile elements that were fused during evolution.

## Results

**Figure 1:**
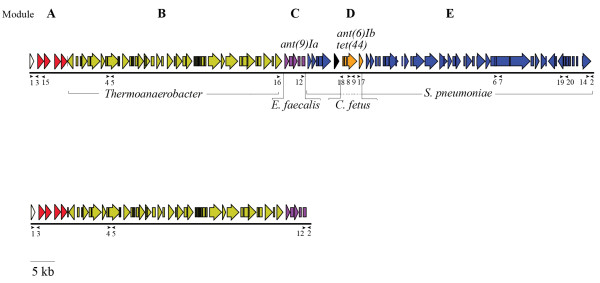
**Schematic view of full Tn *6164 *(top panel) and half the element (bottom panel) and its open reading frames, flanked by *C. difficile *regions.** Various parts of the insert are colored according to their homology. White, ***C***. *difficile*; Red, Module **A**; Yellow, Module **B**; Purple, Module **C**; Orange, Module **D**; Blue, Module **E**; black, unknown. Location of the oligonucleotides used for the data in Table 2 is indicated by arrowheads

Previously, an insert, unique for *C. difficile*, was described in the genome of strain M120, a PCR ribotype 078 strain, isolated from an Irish diarrheic patient [5]. We analyzed the open reading frames (ORFs) present in the insert to investigate their nature and origin (see Figure 1 and Table 1). 

**Table 1:** **Open reading frames encoded by Tn *6164 ***

**Gene**	**Position on Tn *6164***	**Module**	**Sequence identity to**	**Annotation**	**Gene**	**Position on Tn *6164***	**Module**	**Sequence identity to**	**Annotation**
*Orf1*	650-1930	A	-	putative modification methylase	*Orf25*	26793-27122	B	-	conserved hypothetical protein
*Orf2*	1915-3186	A	-	putative modification methylase	*Orf26*	27189-28451	B	*Thermoanaerobacter sp.*	HK97 family phage portal protein
*Orf3*	3252-3962	A	-	hypothetical protein	*Orf27*	28448-29128	B	*Thermoanaerobacter sp.*	Peptidase S14, ClpP
*Orf4*	3952-5031	A	-	ATPase associated with various cellular activities	*Orf28*	29140-30339	B	*Thermoanaerobacter sp.*	HK97 family phage major capsid protein
*Orf5*	5047-6312	A	-	LlaJI restriction endonuclease	*Orf29*	30585-30899	B	*Thermoanaerobacter sp.*	uncharacterized phage protein
*Orf6*	C 7557-6361	A	-	Protein with unknown function, contains a C-terminal CGNR Zinc finger motif	*Orf30*	30903-31238	B	*Thermoanaerobacter sp.*	phage head-tail adaptor, putative
*Orf7*	8000-8494	B	*Thermoanaerobacter sp.*	ECF RNA polymerase sigma-24 factor	*Orf31*	31252-31662	B	*Thermoanaerobacter sp.*	HK97 family phage protein
*Orf8*	8809-9126	B	*Thermoanaerobacter sp.*	rRNA biogenesis protein rrp5, putative	*Orf32*	31659-32012	B	*Thermoanaerobacter sp.*	Protein of unknown function (DUF806);
*Orf9*	9123-10250	B	*Thermoanaerobacter sp.*	Phage associated protein	*Orf33*	32016-32618	B	*Thermoanaerobacter sp.*	DUF3647 Phage protein (HHPred)
*Orf10*	10256-10816	B	*Thermoanaerobacter sp.*	phage-associated protein	*Orf34*	33330-35786	B	*Thermoanaerobacter sp.*	Phage tape measure protein
*Orf11*	10813-12747	B	*Thermoanaerobacter sp.*	DNA-directed DNA polymerase	*Orf35*	35800-36573	B	*Thermoanaerobacter sp.*	phage putative tail component
*Orf12*	12795-13625	B	*Thermoanaerobacter sp.*	Prophage antirepressor	*Orf36*	36692-39100	B	*Thermoanaerobacter sp.*	phage minor structural protein
*Orf13*	13629-14048	B	*Thermoanaerobacter sp.*	DUF 4406 (HHPred)	*Orf37*	39320-39901	B	*Thermoanaerobacter sp.*	Putative Sipho Phage tail protein (HHPred)
*Orf14*	14045-16390	B	*Thermoanaerobacter sp.*	virulence-associated E protein	*Orf38*	39928-42369	B	*Thermoanaerobacter sp.*	glycosyl hydrolase-like protein
*Orf15*	16910-18259	B	*Thermoanaerobacter sp.*	SNF2-related protein	*Orf39*	42430-42855	B	*Thermoanaerobacter sp.*	toxin secretion/phage lysis holin
*Orf16*	18264-18722	B	*Thermoanaerobacter sp.*	phage-associated protein	*Orf40*	42855-43556	B	*Thermoanaerobacter sp.*	N-acetylmuramoyl-L-alanine amidase
*Orf17*	18842-19201	B	*Thermoanaerobacter sp.*	HNH endonuclease	*Orf41*	43975-45540	B	*Thermoanaerobacter sp.*	phage integrase family site-specific recombinase/resolvase
*Orf18*	19314-19865	B	*Thermoanaerobacter sp.*	Phage terminase, small subunit	*Orf42*	45541-45954	B	*Thermoanaerobacter sp.*	recombinase/integrase
*Orf19*	19883-21058	B	*Thermoanaerobacter sp.*	S-adenosylmethionine synthetase	*Orf43*	46222-47529	B	*Thermoanaerobacter sp.*	phage integrase family site-specific recombinase
*Orf20*	21039-22283	B	*Thermoanaerobacter sp.*	DNA methylase N-4/N-6 domain-containing protein	*Orf44*	47987-48856	C	*E. faecalis* pEF418	Nucleotidyl transferase
*Orf21*	22384-23076	B	*Thermoanaerobacter sp.*	hypothetical/virulence-related protein	*Orf45*	48837-49571	C	*E. faecalis* pEF418	methyltransferase
*Orf22*	23445-24344	B	*Thermoanaerobacter sp.*	Putative amidoligase enzyme	*Orf46*	49604-50467	C	*E. faecalis* pEF418	putative aminoglycoside 6-adenylyltansferase
*Orf23*	24382-24843	B	*Thermoanaerobacter sp.*	AIG2/GGCT-like protein	*Orf47*	50511-51038	C	*E. faecalis* pEF418	putative adenine phosphoribosyltransferase
*Orf24*	25462-26685	B	*Thermoanaerobacter sp.*	phage terminase	*Orf48*	51251-51979	C	*E. faecalis* pEF418	putative spectinomycin/streptomycin adenyltransferase
*Orf49*	52403-53176	E	*S. pneumoniae*	phage protein/replication initiator	*Orf71*	77648-79216	E	*S. pneumoniae*	putative surface protein
*Orf50*	53176-54000	E	*S. pneumoniae*	DNA replication protein	*Orf72*	79231-80088	E	*S. pneumoniae*	putative bacteriocin
*Orf51*	53993-54478	E	*S. pneumoniae*	DUF 3801	*Orf73*	80162-80773	E	*S. pneumoniae*	Predicted transcriptional regulator
*Orf52*	54475-55209	E	*S. pneumoniae*	phage antirepressor protein	*Orf74*	80766-81749	E	*S. pneumoniae*	Protein with unknown function
*Orf53*	55202-56890	E	*S. pneumoniae*	TraG/TraD family protein	*Orf75*	82268-82621	E	*S. pneumoniae*	transcriptional regulator, ArsR family
*Orf54*	57454-58486	E	-	DUF 318 Predicted Permease (HHPred)	*Orf76*	82696-83940	E	*S. pneumoniae*	major facilitator superfamily MFS_1
*Orf55*	59048-59398	D	*C. fetus*	glyoxalase family protein	*Orf77*	83927-84403	E	*S. pneumoniae*	toxin-antitoxin system, toxin component, GNAT domain protein
*Orf56*	59411-59938	D	*C. fetus*	transcriptional regulator	*Orf78*	84758-86491	E	*S. pneumoniae*	DNA topoisomerase III
*Orf57*	59988-61910	D	*C. fetus*	tetracycline resistance protein	*Orf79*	86484-87449	E	*S. pneumoniae*	possible DNA (cytosine-5-)-methyltransferase
*Orf58*	62225-63082	D	*C. fetus*	aminoglycoside 6-adenylyltransferase (AAD(6)	*Orf80*	87436-95079	E	*S. pneumoniae*	superfamily II DNA and RNA helicase
*Orf59*	63575-64348	E	*S. pneumoniae*	replication initiator/phage	*Orf81*	95123-95779	E	*S. pneumoniae*	putative single-stranded DNA binding protein
*Orf60*	64345-65172	E	*S. pneumoniae*	replicative DNA helicase	*Orf82*	95939-96841	E	*S. pneumoniae*	transcriptional regulator, XRE family
*Orf61*	65314-65814	E	*S. pneumoniae*	TnpX site-specific recombinase family protein	*Orf83*	97071-98282	E	*S. pneumoniae*	transporter, major facilitator family/multidrug resistance protein 2
*Orf62*	65938-66399	E	*S. pneumoniae*	flavodoxin	*Orf84*	C 99739-98462	E	*S. pneumoniae*	relaxase/type IV secretory pathway protein VirD2
*Orf63*	66817-67302	E	*S. pneumoniae*	putative conjugative transposon protein	*Orf85*	C 101169-99795	E	*S. pneumoniae*	conjugal transfer relaxosome component TraJ
*Orf64*	67299-68033	E	*S. pneumoniae*	phage antirepressor protein	*Orf86*	C 101403-100321	E	*S. pneumoniae*	toxin-antitoxin system, toxin component, Fic family
*Orf65*	68026-69816	E	*S. pneumoniae*	TraG/TraD family protein/putative conjugal transfer protein	*Orf87*	C 101878-101396	E	*S. pneumoniae*	putative membrane protein
*Orf66*	70395-70706	E	*S. pneumoniae*	putative single-strand binding protein	*Orf88*	C 102435-101887	E	*S. pneumoniae*	putative toxin-antitoxin system, toxin component
*Orf67*	70934-71797	E	*S. pneumoniae*	conjugative transposon membrane protein	*Orf89*	C 102845-102444	E	*S. pneumoniae*	regulator/toxin-antitoxin system, antitoxin component
*Orf68*	72099-72509	E	*S. pneumoniae*	conjugative transposon membrane protein	*Orf90*	103034-103555	E	*S. pneumoniae*	conserved hypothetical protein
*Orf69*	72580-74823	E	*S. pneumoniae*	type IV conjugative transfer system protein	*Orf91*	103825-104235	E	*S. pneumoniae*	sigma-70, region 4
*Orf70*	74831-77410	E	*S. pneumoniae*	conjugative transposon cell wall hydrolase/NlpC/P60 family	*Orf92*	104966-106712	E	*S. pneumoniae*	site-specific recombinase, resolvase family

### The 100 kb insert has a modular composition

Bioinformatic analysis revealed that the insert has a modular composition. The 3’ end of the insert (module E) is homologous to Tn*1806* of *S. pneumoniae* which confers erythromycin resistance. Although this element has not been shown to transfer via conjugation, transfer via transformation was shown [22]. In *C. difficile* strain M120 this element appears to be the backbone into which several other elements have been inserted (see Figure 1 top panel). The first 7.3 kb on the 5’ end of the insert (module A) has only moderate homology (60–70% maximum sequence identity) to known sequences. Interestingly, this part of the insert contains 2 putative modification DNA methylases and a putative endonuclease, possibly enabling a form of molecular vaccination as described by Kobayashi et al. [23]. During this process methylation protects the incoming element from host endonucleases and, following integration, will protect the host chromosome from endonucleases present on other mobile genetic elements. This sequence is followed by a complete prophage of approximately 39.5 kb (module B), which shows 92% sequence identity to a *Thermoanaerobacter* sp. prophage (Genbank accession no. CP002210). The next 4.5 kb stretch (module C) is 99% identical to part of the *Enterococcus faecalis* plasmid pEF418 containing, amongst others, a putative methyltransferase and a putative spectinomycin adenyltransferase (*ant(9)Ia*) [24]. It is also described to be part of a pathogenicity island in *Streptococcus suis*[25]. Finally, an insertion of approximately 4.5 kb (module D) with 90% sequence identity to the transferable pathogenicity island of *Campylobacter fetus* subsp *fetus*[26] is present within the sequence of Tn*1806*. This sequence contains, amongst others, putative *tet*(44) and *ant(6)-Ib* genes, which could respectively confer tetracycline and streptomycin resistance.

The G + C content of the entire insert (34%) was significantly higher than that of the entire genome (29%), clearly indicating that the insert was of foreign origin (see Additional file 1). In addition, within the insert the different modules could be distinguished by their G + C contents. The G + C content of module A, B, C, D and E was 31%, 41%, 35%, 28% and 31%, respectively.

### The 100 kb insert is a transposon

Based on the bioinformatic comparison of the insert described above, the possible excision of 3 (independent) elements was predicted. Primers were designed (primers 14–20, see Table 3) to amplify the circular intermediates of the complete insert (primers 14 and 15)*,* the putative *Thermoanaerobacter* sp. phage (module B, primers 15 and 16) and the *C. fetus* pathogenicity island (module D, primers 17 and 18) of the element. PCR confirmed only the excision and circularisation of the entire insert (results not shown). It is expected that the serine recombinase at the 3’ end of the element is responsible for excision (see Table 1). Sequencing of the circular intermediate was used to determine the precise ends of the element, showing the element is flanked by a TG dinucleotide; serine recombinases prefer a 2 bp crossover site identical in the target site and joint of the circular intermediate [27]. *In silico* extraction of this sequence from the genome confirms that the element is present in the homologous target site of CTn*2* in strain 630 [7]. The precise size of the element is 106,711 bp and it runs from bp 418,525-525,236 (including the TG dinucleotide at both ends) in the M120 genomic sequence (GenBank accession no. FN665653). Upon our request, the transposon number Tn*6164* was provided by the Transposon registry [28] (http://www.ucl.ac.uk/eastman/tn/index.php).

To test the conjugative transfer of the element, filter mating assays were performed, selecting for the possible tetracycline resistance by means of the *tet*(44) gene. However, M120 contains also a copy of *tet*(M) present on a conjugative transposon with 97% sequence identity to Tn*916*[16], which we have designated Tn*6190*. This element has inserted intragenically in the homologue of *C. difficile* strain 630 ORF CD2015. Tn*6190* contains homologues to all Tn*916* ORFs except *orf*12 which is involved in regulation of *tet*(M) through transcriptional attenuation [29].

During filter mating experiments with M120 as a donor strain and CD37 as a recipient, all putative transconjugants were identified as the recipient strain. In total 70 transconjugants were tested by PCR, using primers Lok1, Lok3 [13],[19,20], Tn*916* Fw, and Tn*916* Rev [30]. However, none contained Tn*6164,* all contained only Tn*6190* (results not shown).

### Tn*6164* is sporadically present in PCR ribotype 078

Simultaneously with the publication of the M120 sequence, we obtained Illumina sequence reads of the *C. difficile* strain 31618, which was isolated from a diarrheic piglet from a pig farm in the Netherlands [16]. Comparative genomic analysis of 31618 to M120 revealed an almost complete overlap of the two genomes. However, reference assembly of the 31618 reads to M120 showed that Tn*6164* was not present in 31618 (results not shown). This prompted us to investigate the prevalence of Tn*6164* in PCR ribotype 078 strains. We designed a PCR to show presence (primers 1 and 3) or absence (primers 1 and 2) of Tn*6164* in PCR ribotype 078 genomic DNA (see Figure 1 top panel). In addition, in view of the heterogeneous origin of Tn*6164* and to investigate the presence of both the *Thermoanaerobacter* prophage and *Streptococcus* DNA (Modules B and E, respectively), we designed two more PCRs (primers 4–5 and 6–7). Finally, we designed a PCR to detect the presence of the *tet*(44) gene present on Tn*6164* (Module D, primers 8 and 9). Besides the sequenced 31618 strain, 173 human PCR ribotype 078 strains and 58 porcine PCR ribotype 078 strains (from 27 pig farms) were tested for the presence of these elements.

A minority of the isolates tested did contain a DNA insert at the indicated location in the genome; 18 of the 231 isolates (7.8%) were positive in the 1–3 PCR (Table 2). Remarkably, all 18 strains were tetracycline resistant human isolates. None of the porcine strains contained an insert at the position tested. Strains positive in the 1–3 PCR were negative in the 1–2 PCR, and *vice versa*, showing complete complementarity of the two PCRs in PCR ribotype 078 strains.

**Table 2:** Detection of specific regions of Tn6164 in PCR ribotype 078 strains

**Strain**	PCR 1-2^*^	**PCR1-3**^**§**^	**PCR 4-5**^**#**^	**PCR 6-7**	**PCR 8-9**^**†**^	**PCR 12-2‡**
56/69	-	+	+	-	-	+
26222	-	+	+	-	-	+
26114	-	+	+	-	-	+
26247	-	+	+	-	-	+
26235	-	+	+	-	-	+
ES1203	-	+	+	-	-	n.t.
6065935	-	+	+	-	n.t.	n.t.
7047337	-	+	+	-	n.t.	n.t.
8088158	-	+	+	-	n.t.	n.t.
50/19	-	+	+	+	+	-
GR0106	-	+	+	+	+	n.t.
DE1210	-	+	+	+	+	n.t.
BG1209	-	+	+	+	+	n.t.
NO1311	-	+	+	+	+	n.t.
NO1307	-	+	+	+	+	n.t.
IE1102	-	+	+	+	+	n.t.
GR0301	-	+	+	+	+	n.t.
10053737	-	+	+	+	n.t.	n.t.

^*^PCR only positive when no insert is present, ^§^PCR only positive when insert is present ^#^PCR detects Module B, ^¶^PCR detects module E, ^†^PCR detects module D. ‡ PCR only positive in strains containing half of the element. Location of the oligonucleotides used is indicated in Figure 1. +, PCR positive; -, PCR negative; n.t., not tested.

### Evidence for multiple insertions in Tn*6164*

All the strains that contained an insert (based on the 1–3 PCR) were further analyzed for the presence of Module B and E present in Tn*6164*, using primer pairs 4–5 and 6–7 (see Figure 1 top panel and Table 3). Only nine of 18 strains positive for PCR 1–3 were positive for PCRs 4–5 and 6–7, suggesting the presence of the complete element as described for M120. The other 9 strains were only positive for Module B (PCR 4–5), showing the existence of alternative (shorter) elements (see Table 2), as predicted by the bioinformatic analysis. The strains that were positive for Module E (PCR 6–7) were also positive for Module D (PCR 8–9, see Table 2). In contrast, strains containing Module B, but not Module E, thus containing only half the element, also lacked Module D. This indicates that the 3’end of half the element was situated upstream of Module D.

**Table 3:** Oligonucleotides used in this study

**Name**	**Sequence**	**Purpose**
1	GAGATATGGTTATGAGATTAGG	Presence/absence of insert
2	CCCACCTTTATAGCATCATATAG	Absence of insert
3	CTAACCTATCAACTCAACCCC	Presence of insert
4	AGGATAAGACCGCAGCAGAA	Presence 5’half of insert
5	AAAAACGACGGTTTTCCTGTG	Presence 5’half of insert
6	GGGCAAATAGAAAGTCAAAACG	Presence 3’half of insert
7	AAGTGGTGTTTTCTTTGGAGGA	Presence 3’half of insert
8	CCACAGGGATACCTTTCTCGTGC	Presence of *tet*(44) gene
9	TTCCATATCCTCGGGTTTTTGCAT	Presence of *tet*(44) gene
10	CAGGTGTTGAAATAGATATTGAG	Detect 3' end half insert
11	CAGAAGTCGATCCTTTCTGGG	Detect 3' end half insert
12	GGTGGCTGAACTCGTTAATC	Detect 3' end half insert
13	CTCCACATGGCTCGAGTTG	Detect 3' end half insert
14	GAGGAATTTAACAGAACAGTATTT	Excision studies
15	TCTATCCTGCCTTCTCAACC	Excision studies
16	CGAATCGCTGAAATGACTGA	Excision studies
17	GCGAATGATTTCATGGAAGG	Excision studies
18	CGACTGCATTACCAGTTCCA	Excision studies
Lok1 [13]	AAAATATACTGCACATCTGTATAC	Transconjugant screening
Lok3 [13]	TTTACCAGAAAAAGTAGCTTTAA	Transconjugant screening
19	CAGCTGCAGTTTTTCCATGA	Transconjugant screening
20	GCAGCTAACGGTGATGACAA	Transconjugant screening
Tn*916* Fw [30]	GACGGAAGATACTTATACA	Transconjugant screening
Tn*916* Rev [30]	GCCTTTGGATTCATTCCTGC	Transconjugant screening

Of the isolates that were only positive for the PCR 4–5, the exact 3’end of the insert was determined by sequencing the PCR product obtained with primers 12 and 2 (see Figure 1 bottom panel), which yielded a 350 bp product. The border of the 3’end was between the 3’ end of Module C and the 5’end of Module E. A similar sequence was found at the homologous site when the full element was present, but also at the 3’ end of the full element, the 5’ end of the element, the joint of the circular intermediate and the predicted target site as based on the 630 sequence (see Table 4). This indicates that Tn*6164* was created by two elements integrating in the same target site (next to each other) and fusing, with a second copy of the target site still present between the two original elements within Tn*6164*.

**Table 4:** **Sequences of the joints between the genome and Tn *6164 *and the joint of the circular form **

	
CGCATTGCG-AGACTATAG	3’ends of half insert
CGCATTGCG-AGACTATAG	3’ends of full insert
CTCA-TGTGGAGTGCGTGG	5’end of full insert
GCCA-TGTGGAGACTATAG	middle section of full element
CACA-TGCGTTGTCTTGTG	Joint of circular intermediate Tn*6164*
CACATTGTG-AGACTGTAG	CTn*2 *target site in strain 630

### Absence of Tn*6164* sequences in other PCR ribotypes

Since PCR ribotype 126 has been shown to be very closely related to PCR ribotype 078, with an almost indistinguishable PCR ribotype banding pattern, we also tested a small collection of PCR ribotype 126 strains with the 1–2 and 1–3 PCRs. In none of the 10 PCR ribotype 126 strains tested could we demonstrate the presence of an insert at the site in which Tn*6164* was inserted in M120 (results not shown).

In addition, a collection of 66 other PCR ribotypes was tested as well. This collection consisted of the 25 most frequently found PCR ribotypes in Europe, supplemented with the Leeds-Leiden collection [31]. None of the other PCR ribotypes, was positive for PCR 1–3, 4–5 or 6–7.

### No antibiotic resistance phenotype linked to presence of Tn*6164*

Since several putative antibiotic resistance genes were found to be present on the element (see Figure 1 and Table 1), strains containing full Tn*6164*, only half of the element, or no element at all were tested for antibiotics resistance. Resistance to tetracycline, spectinomycin and streptomycin was tested using several methods (see materials and methods). Surprisingly, no correlation was found between the presence of *tet*(44), *ant(6)Ib* or *ant(9)Ia* and resistance to tetracycline, spectinomycin or streptomycin (see Table 5).

**Table 5:** Antibiotic sensitivity of PCR ribotype 078 strains with.doc

**Genes present (transposon)**	**Strain**	**MIC Tet (μg/ml)**	**MIC Spec (μg/ml)**	**Strep**
	56/69	24	> 750	N.D.
	26222	16	N.D.	R
*ant(9)Ia* (Tn*6164*)	26114	32	N.D.	R
*tet*(M) (Tn*6190*)	26247	16	> 750	R
	26235	48	N.D.	N.D.
	06065935	8	N.D.	R
	50/19	48	>750	S
	GR0106	12	>750	R
*ant(9)Ia* (Tn*6164*)	DE1210	8	>750	R
*ant(6)* (Tn*6164*)	BG1209	8	>750	R
*tet*(44) (Tn*6164*)	NO1311	12	>750	R
*tet*(M) (Tn*6190*)	NO1307	8	>750	R
	IE1102	12	>750	R
	GR0301	8	>750	R
	10053737	N.D	N.D	R
*tet*(M) (Tn*6190*)	45/22	8	>750	N.D.
	29/74	<8	>750	N.D.
	31618	N.D.	<250	N.D.
None	07053152	<8	N.D.	R
	R20291(027)	N.D.	<250	N.D.

R, resistant (no halo around diffusion disk); S, sensitive (15 mm halo).

### Strains containing full Tn*6164* are all genetically related

**Figure 2:**
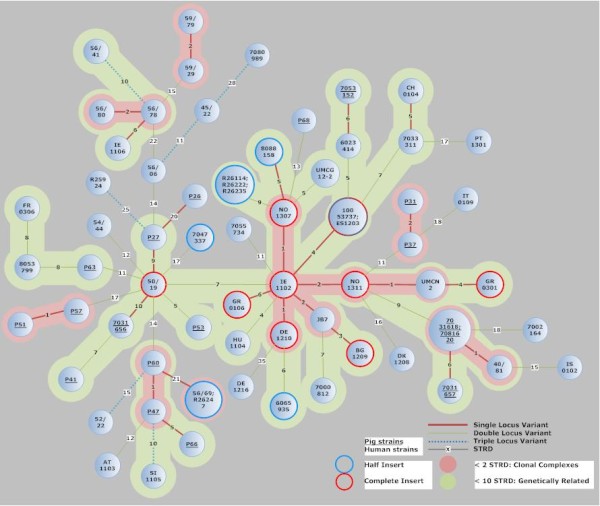
**Minimum spanning tree of all the PCR ribotype 078 isolates that contained an insert (50 or 100 kb), supplemented with strains not containing the element.** Each circle represents either one unique isolate or more isolates that have identical MLVA types. Red circles indicate strains with full Tn*6164* and blue circles indicate strains with half the element. The numbers between the circles represent the summed tandem-repeat differences (STRD) between MLVA types. Underlined numbers represent porcine strains and normal numbers represent human isolates. Thick red lines represent single-locus variants; thin green lines represent double-locus variants and dotted blue lines represent triple locus variants between MLVA types. Clonal clusters are defined by an STRD of <2 (pink area), and genetically related clusters are defined by an STRD of <10 (green area)

Since we could not find many isolates containing Tn*6164*, we reasoned that the element could be relatively recently acquired and that the isolates thus might be genetically closely related. Therefore, we applied MLVA [3,16] on all the isolates containing Tn*6164*, or only half of it, supplemented with a number of isolates without the element, to investigate the genetic relatedness of the strains. In Figure 2, a minimal spanning tree of all the isolates containing an element is shown, with control strains. Based on the MLVA, all the isolates containing full Tn*6164* (n = 9) are genetically related (STRD < 10) and four of them are in one clonal complex. Six isolates containing half of the element are also in this genetically related cluster, whereas the other three isolates containing half the element are not (STRD > 10). 

### Suggestive link between the 100 kb insert and increased virulence

To investigate a possible increased virulence of strains containing the element, clinical parameters of patients with a *C. difficile* infection due to a strain that contained Tn*6164* were compared to parameters of patients that suffered from a strain that did not contain the full element. Patients with Tn*6164* resembled patients without the element concerning demographic characteristics. Clinical characteristics were only known for patients from the ECDIS study [32] and patients registered in the CDRL (n = 84). Patients with and without the element suffered from severe diarrhea in similar proportions. Mortality due to CDI was more common in patients infected with *C. difficile*::Tn*6164* (29% *vs* 3%). This suggests that Tn*6164* might convert PCR ribotype 078 strains to a more virulent strain. However, since the number of patients infected with a Tn*6164*-positive strain, and for which the clinical data was available, was very low (n = 7), no multivariate analysis could be performed, which means that a bias cannot be ruled out. Further research is needed to confirm a possible link between increased virulence and the presence of Tn*6164*.

## Discussion

PCR ribotype 078 has recently emerged as a hypervirulent *C. difficile* strain [2,3]. Previously published MLVA studies have shown that all PCR ribotype 078 strains are closely related [3], irrespective of human or porcine origin [16], fostering the notion that PCR ribotype 078 infection could be a zoonosis. Recently, the full genome sequence of a *C. difficile* PCR ribotype 078 strain was published [5]. This M120 strain was shown to contain a unique insert of approximately 100 kilobases. In this paper we show that this insert is a transposable element, Tn*6164*. It is not representative for all PCR ribotype 078 strains. On the contrary, we found that the majority of the PCR ribotype 078 strains do not contain the element. Moreover, some strains contain only half of the element. So, three different kinds of PCR ribotype 078 can now be distinguished: Those with a full length element, those with half the element, and those with no element at all. Tn*6164* was exclusively found in tetracycline resistant PCR ribotype 078 strains, isolated from humans. We tested a collection of other PCR ribotypes, of which none contained the element. Since we only tested 1 strain per PCR ribotype, we cannot rule out the possibility that Tn*6164* is present in other PCR ribotypes. We covered the whole genomic spectrum of *C. difficile* since we tested multiple samples of each genetic clade previously identified [10,33-35]. In addition, Tn*6164* has not been found in any other *C. difficile* genome that has been published so far than M120.

Although Tn*6164* contained a *tet*(44) gene, we could not demonstrate increased tetracycline resistance of strains containing the element. Previously, it has been shown that this gene, present on a homologues resistance island, is active in *C. fetus*[26]. In *C. difficile*, the copresence of the *tet*(44) gene on Tn*6164* and the *tet*(M) gene on the Tn*6190* in one bacterium does not result in an increased resistant phenotype. Also the spectinomycin and streptomycin resistance genes did not result in a phenotype, despite the presence of two potential aminoglycoside resistance genes (*ant(9)Ia)* and *ant(6)*) on Tn*6164* (see Figure 1 and Table 1). We do not know if the resistance genes are expressed in M120. However, since we show the presence of the circular intermediate transposon DNA, some activity of transposon related genes is expected.

Since we have only found Tn*6164* in strains also containing Tn*6190*, it is possible that Tn*6164* transfer is dependent on Tn*6190*. Further research is needed to investigate the possibility of Tn*6190*-dependent transfer of Tn*6164*. In addition, remarkably, Tn*6164* (the whole or half the element) was significantly (p = 0.01) more found in strains isolated from humans than in strains isolated from pigs. Although the same strains circulate in humans and pigs [16], and also Tn*6190* circulates in pig strains [16], we did not find any porcine strain that contained the element. We have no explanation for this difference.

None of the transconjugants tested showed the presence of Tn*6164*, but all contained Tn*6190*. These results indicate that Tn*6164* has a (much) lower transfer frequency than Tn*6190.* Nevertheless, a complete set of proteins, required for transfer, is present on Tn*6164*. Loss of Tn*6190* or introduction of another selection marker in Tn*6164*[11] could prove to be a strategy to further study the capability of conjugative transfer of this element.

Tn*6164* has integrated intergenically between homologs of the 630 ORFs CD0406 and CD0437, a tRNA methyltransferase and a hypothetical protein respectively. In strain 630, this target site is occupied by the conjugative transposon CTn*2*[7,11]. There is no significant homology between Tn*6164* and CTn*2*. The empty target site is present in many sequenced strains of *C. difficile.* However, no other mobile genetic elements have been reported to integrate at this site.

It was impossible to phenotypically distinguish strains containing Tn*6164* from strains without the element. Although we have no transcriptional data available of the genes that are located on Tn*6164* it is clear that it could provide an advantage under certain circumstances. In this respect it is interesting to note that the patients suffering from an element-containing strain are suggested to undergo a more severe illness than patients with a strain not containing Tn*6164*. However, because of the low number of strains containing the insert no multivariate analysis could be carried out. Therefore, we cannot rule out that these data are biased. Further research is needed to confirm this observation.

Isolates containing the full element originated from all over Europe, including Ireland, England, Norway, Germany, Bulgaria, Greece and the Netherlands, whereas isolates containing half the element were only found in the United Kingdom, Spain and the Netherlands. MLVA showed genetic relatedness between most of the strains, although no epidemiologic link between the strains from different countries could be found. It has recently been shown that PCR ribotype 078 strains show a lot less heterogeneity in MLVA than for instance PCR ribotype 027 or PCR ribotype 017 [36-38]. This could indicate a higher level of relatedness, or it could mean that the mechanism behind the MLVA variability is different in PCR ribotype 078 strains than in other PCR ribotypes [16].

Altogether, we show the presence of a 100 kb transposon in some *C. difficile* PCR ribotype 078 strains. Although we could not show any evolutionary benefits of the transposon, it could very well serve as a reservoir of antibiotic resistance [26], for commensal bacteria in the human gut.

## Conclusions

Tn*6164* is a novel transposon of approximately 100 kb, found sporadically in *Clostridium difficile* PCR ribotype 078 strains, isolated from humans. Tn*6164* has a modular composition and is the product of multiple insertions of separate elements from various origins, as evidenced by the existence of strains containing only half the element. Strains containing Tn*6164* were all genetically related. We were not able to find a readily distinguishable phenotype for strains containing the element, although several potential antibiotic resistance genes were present on Tn*6164*. Tn*6164* may act as a source of antibiotic resistance genes in the human gut. Further research is needed to investigate if Tn*6164* plays a role in the virulence of PCR ribotype 078 *Clostridium difficile* strains.

## Methods

### Bacterial Isolates and culture conditions

PCR ribotype 078 *C. difficile* strain 31618 was obtained from a pig farm in the eastern part of the Netherlands where neonatal diarrhea was present. Culturing of the feces yielded *C. difficile*, as determined by an in-house PCR for the presence of the *gluD* gene encoding the glutamate dehydrogenase specific for *C. difficile*[39]. PCR ribotype was determined as previously described [40].

The other PCR ribotype 078 strains used in this study were obtained from a previously described PCR ribotype 078 strain collection [16], consisting of strains isolated from humans and pigs, supplemented with human PCR ribotype 078 strains from the ECDIS (European *Clostridium difficile* Infection Survey) study in 2010 [32]. In addition, recently isolated PCR ribotype 078 strains from Dutch diarrheic piglets (2007–2010) and human (2006–2010) strains collected by the Dutch *C. difficile* Reference Laboratory (CDRL) were used. The 58 Pig strains were collected on 27 pig farms in the Netherlands.

PCR ribotype 126 strains used in this study originate from the ECDIS study, isolated in 2010, from several countries in Europe [32]. PCR ribotype reference strains (n = 68) were obtained from the CDRL.

The nontoxinogenic strain CD37 [41,42] was used as a recipient in filter mating experiments as this has previously been shown to be a good recipient for mobile genetic elements from other *C. difficile* strains [11].

*C. difficile* strain M120 was kindly provided by Dr. Trevor Lawley (Sanger Institute). Standard culturing of *C. difficile* isolates was carried out on blood agar plates at 37°C and anaerobic conditions.

### DNA Sequencing, reference assembly and annotation

DNA was isolated from one colony of the 31618 strain by standard techniques [43]. The isolate was sequenced using the Illumina platform (Solexa) at the Leiden Genome Technology Center (LGTC) at the LUMC, using the manufacturers’ protocols. Single end reads were generated and submitted to the NCBI sequence read archive (http://www.ncbi.nlm.nih.gov/sra) under accession number SRX030155. A reference assembly of the reads was carried out against strain *C. difficile* PCR ribotype 078 strain M120 (GenBank accession no. FN665653), using CLC genomics workbench (CLCbio, Aarhus, Denmark). Number of reads used was 5267302, of which 2968638 reads could be mapped to the M120 genome sequence. The unique 100 kb insert present in M120 was readily identified with the CLC genomics workbench. The ORFs present in the insert were identified by CLC genomics workbench and annotation was carried out manually, using BLAST and SMART. ORFs identified as “protein of unknown function” were further analyzed by profile-profile searches through HHpred (http://toolkit. tuebingen.mpg.de/hhpred).

### Bioinformatic comparison of the mixed origin of Tn*6164*

The genome of strain M120 was compared to the genomes of *C. difficile* 630 (Genbank accession no. AM180355), *Thermoanaerobacter* sp. (GenBank accession no. CP002210), *S. pneumonia* (Genbank accession no. CP002121) and *C. fetus* (Genbank accession no. FN594949) using the Artemis Comparison Tool [44].

### Circularization of the transposon

In order to investigate if the putative element could excise itself from the genome, PCR analysis was performed to amplify the joint region of a circular molecule using primers at the ends of the element, facing outward (primers 14 and 15 in Table 3). PCR amplifications were carried out using the NEB *Taq* Polymerase kit (New England Biolabs, Herts, UK) according to the manufacturer’s instructions with 10 mM dNTPs (NEB). The primers that were used are listed in Table 3 (Sigma-Genosys, UK).

### Filter-matings assays

Filter-matings were carried out as described previously [45]. *C. difficile* strains M120 and CD37 were cultured on Brain heart infusion (BHI) (Oxoid Ltd.) agar supplemented with 5% Horse blood (E&O laboratories). *C. difficile* strain CD37 was used as recipient. Transconjugants were selected for on BHI plates supplemented with 25 μg/ml rifampicin (Sigma Aldrich) and 10 μg/ml tetracycline (Sigma Aldrich). Transconjugants were examined using PCR with primer pair Lok1/Lok3 to confirm identity of the recipient strain and primer pairs Tn*6164* accessory region Fw + Rev and Tn*916* Fw + Rev to confirm the transfer of Tn*6164* or Tn*6190*.

### Inverse PCR

*C. difficile* genomic DNA was digested with PstI or EcoRI. After purification, the genomic DNA fragments were self-ligated to create circular DNAs. Subsequently, the DNA was precipitated and dissolved in H_2_O. PCR was carried out on the DNA, using primers 4-rev and 5-rev or 14 and 15 (annealing at 58°C, 35 cycles). PCR products were visualized by gel electrophoresis and sequences were determined through direct sequencing on the purified PCR amplicons or through cloning into pCR2.1/TOPO (Invitrogen) and subsequent sequencing with the plasmid-located primers T7 and M13 reverse.

### Antibiotic resistance

The MIC for tetracycline was determined using E tests (BioMérieux, Boxtel, the Netherlands) on blood plates under anaerobic conditions at 37°C. Breakpoint for tetracycline was 8 μg/ml. Spectinomycin resistance was determined by an agar dilution method of *C. difficile* colonies on BHI agar plates, supplemented with increasing amounts of spectinomycin. Streptomycin resistance was tested by disk diffusion method, using Sensi-Neotabs (Rosco, Denmark) (Streptomycin 500 ug disks) on blood plates under anaerobic conditions at 37°C.

### Oligonucleotides

Oligonucleotides used in this study are shown in Table 3.

### PCR

PCRs were carried out using Gotaq polymerase (Promega, Leiden, the Netherlands). Reactions contained 0.4 mM dNTPs, 0.4 uM oligonucleotides. Annealing temperature of the PCR was set at 50°C and PCRs were standardized at 30 cycles.

### Statistical analyses

Patients samples with the full 100 kb insert were compared to patients samples with a part of the insert or no insert. The Chi-square test and *t*-test were used to calculate the p-value. Analyses were performed using the SPSS for Windows software package, version 17.0.

### MLVA

Sixty eight strains were subjected to MLVA, of which 39 were previously characterized [16]. MLVA and construction of the minimal spanning tree based on the MLVA results were carried out as described previously [16].

## Competing interests

The authors declare that they have no competing interests.

## Authors’ contributions

JC designed the study, carried out PCRs, antibiotic resistance assays, analyzed the data and wrote the paper; DB carried out sequencing and analyzed the data; MB carried out the circularization and filter mating experiments and wrote the paper; CH managed the strain collections and carried out MLVA; MH carried out statistical analysis and wrote the paper; AM carried out filter mating experiments and wrote the paper; LL gathered pig samples; EK designed the study and wrote the paper; HL designed the study, analyzed data and wrote the paper. All authors read and approved the final manuscripts.

## Supplementary Material

Additional file 1 **Circular representation of the genome of * C. difficile * strain M120.**The two concentric circles represent the genome (outer circle) and the G + C content (inner circle; window size 10,000; Step size 200). Green represents values higher than average (29%), purple below average. In between the two circles, the presence of the two transposable elements is indicated in red (Tn6164) and blue (Tn6190). Figure was created using DNA plotter [46]. Click here for file
