# Supplementary figures and images for: Analysis of a Clostridium difficile PCR ribotype 078 100 kilobase island reveals the presence of a novel transposon, Tn6164
Source: BMC Microbiol. 2012 Jul 2;12:130. doi: 10.1186/1471-2180-12-130 (PMC3485107; doi:10.1186/1471-2180-12-130)

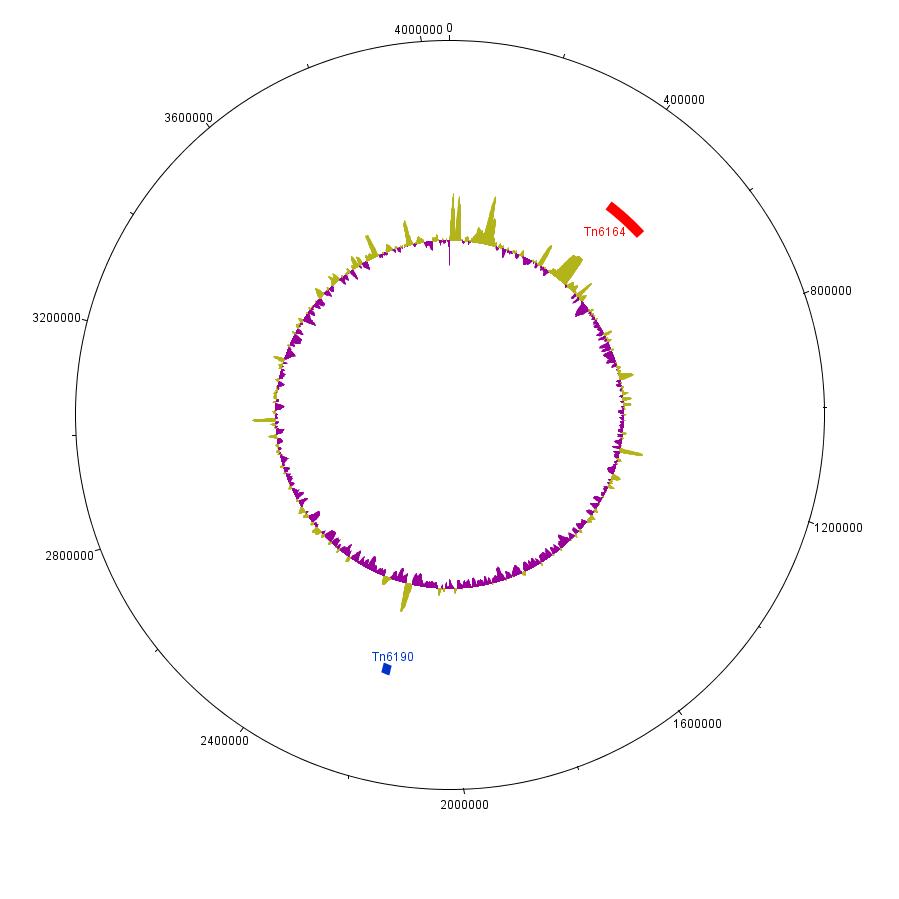

Supplement: Additional file 1 — Circular representation of the genome of C. difficile strain M120.The two concentric circles represent the genome (outer circle) and the G + C content (inner circle; window size 10,000; Step size 200). Green represents values higher than average (29%), purple below average. In between the two circles, the presence of the two transposable elements is indicated in red (Tn6164) and blue (Tn6190). Figure was created using DNA plotter [46]. [file 1471-2180-12-130-S1.jpeg]
